# *Akkermansia muciniphila* Cell-Free Supernatant Improves Glucose and Lipid Metabolisms in *Caenorhabditis elegans*

**DOI:** 10.3390/nu15071725

**Published:** 2023-03-31

**Authors:** Zhong-Qin Wu, Xin-Ming Chen, Hui-Qin Ma, Ke Li, Yuan-Liang Wang, Zong-Jun Li

**Affiliations:** 1 Hunan Province Key Laboratory of Food Science and Biotechnology, College of Food Science and Technology, Hunan Agricultural University, Changsha 410128, China; hnwuzhongqin@163.com (Z.-Q.W.); chenxmyesswl@163.com (X.-M.C.); mbomb0730@163.com (H.-Q.M.); leeke2014@163.com (K.L.); wangyuanliang@hunau.edu.cn (Y.-L.W.); 2 National Research Center of Engineering Technology for Utilization of Functional Ingredients from Botanicals, Changsha 410128, China

**Keywords:** *Akkermansia muciniphila*, postbiotic, *Caenorhabditis elegans*, glucose metabolism, lipid metabolism

## Abstract

To explore the mechanism by which *Akkermansia muciniphila* cell-free supernatant improves glucose and lipid metabolisms in *Caenorhabditis elegans*, the present study used different dilution concentrations of *Akkermansia muciniphila* cell-free supernatant as an intervention for with *Caenorhabditis elegans* under a high-glucose diet. The changes in lifespan, exercise ability, level of free radicals, and characteristic indexes of glucose and lipid metabolisms were studied. Furthermore, the expression of key genes of glucose and lipid metabolisms was detected by qRT-PCR. The results showed that *A. muciniphila* cell-free supernatant significantly improved the movement ability, prolonged the lifespan, reduced the level of ROS, and alleviated oxidative damage in *Caenorhabditis elegans*. *A. muciniphila* cell-free supernatant supported resistance to increases in glucose and triglyceride induced by a high-glucose diet and downregulated the expression of key genes of glucose metabolism, such as *gsy-1*, *pygl-1*, *pfk-1.1*, and *pyk-1,* while upregulating the expression of key genes of lipid metabolism, such as *acs-2*, *cpt-4*, *sbp-1*, and *tph-1,* as well as down-regulating the expression of the *fat-7* gene to inhibit fatty acid biosynthesis. These findings indicated that *A. muciniphila* cell-free supernatant, as a postbiotic, has the potential to prevent obesity and improve glucose metabolism disorders and other diseases.

## 1. Introduction

*Akkermansia muciniphila* (*A. muciniphila*), as a new generation of anaerobic potential probiotics, is widely distributed in the intestinal mucus layer, and uses mucin attached to the intestinal surface as its only source of carbon and nitrogen to catabolize small molecules, promoting intestinal homeostasis [1,2]. In infancy, *A. muciniphila* can be used as a marker of the growth and diversity of intestinal flora. During the process of growth and development, the number of *A. muciniphila* in the intestinal tract increases significantly [3,4]. As a key member of the intestinal microbiota, it plays an important role in maintaining health. Previous studies have shown that a decrease of *A. muciniphila* abundance is related to the development of some diseases, including obesity, diabetes, colitis, etc. [5,6,7] One study reported that feeding live *A. muciniphila* to obese and type 2 diabetic mice reduced fat, metabolic endotoxemia, and insulin resistance, and increased levels of endogenous cannabinoids that control intestinal inflammation, repair intestinal barriers, and secrete intestinal peptides [8]. Therefore, *A. muciniphila* live bacteria have obvious biological activity in regulating glucose and lipid metabolism.

With numerous studies confirming the probiotic effects of live *A. muciniphila* bacteria, attention has been paid to whether their metabolites have similar probiotic effects. A P9 protein secreted by *A. muciniphila* was found to induce the secretion of glucagon-like peptide-1 in vivo after binding to intercellular adhesion molecules, and this mechanism of action effectively promoted insulin levels and adipose tissue catabolism for thermogenesis, producing a prometabolic effect of blood glucose maintenance and reduction of fat accumulation [9]. In addition, *A. muciniphila* metabolites have been reported to regulate host glucolipid metabolism and modulate insulin sensitivity [10]. *A. muciniphila* can use mucin catabolism to produce short-chain fatty acids, which provide an energy source for cells and participate in metabolic pathways, thereby regulating the metabolic levels of the body [11]. Recent studies have further revealed that the active catabolism of mucin by *A. muciniphila* in the intestine to release acetate and propionate, which in turn inhibit the expression of the cholesterol biosynthesis genes Sqle, Hmgcs, and Hmgcr, provide additional health benefits by regulating lipid metabolism [12]. Propionate, one of the metabolites of *A. muciniphila*, can be affected by various metabolic regulators in epithelial cell expression and is involved in lipid metabolism, such as through fasting-induced adipose factor, G protein coupled receptors 43, histone deacetylase 3, and peroxisome proliferator-activated receptor-γ (PPAR-γ) [13]. Another study demonstrated that extracellular vesicles released by *A. muciniphila* reduced intestinal permeability in mice with high-fat diet-induced colitis and induced increased expression of PPAR-α and PPAR-γ mRNA in the adipose tissue of the epididymis [14,15]. Therefore, the metabolites of *A. muciniphila* have postbiotic effects and contribute to the improvement of metabolic disorders. Existing studies have been largely based on the apparent effects in clinical and animal models, but few studies have confirmed a causal relationship; therefore, the specific mechanisms by which *A. muciniphila* metabolites affect metabolic biological processes needed to be further investigated.

*Caenorhabditis elegans* (*C. elegans*) is characterized by a shorter lifespan, lower exercise rates, and increased triglycerides or neuronal defects under a high-glucose diet. It has been recognized as a suitable animal model to explore the mechanisms of glucose and lipid metabolisms [16,17,18,19]. Its whole body is transparent, and it is easy to stain lipid droplets in the subcutaneous and intestinal cells by oil red O for intuitive observation of phenotypes [20]. *C. elegans’* regulation of glucose and lipid metabolisms pathways is highly conservative to humans. For instance, the insulin/IGF-I signaling pathway can regulate the homeostasis of lipid metabolism, and the serotonin pathway can control lipid metabolism and nematode feeding behavior [21]. Therefore, this study took *C. elegans* as an animal model to explore the protective effect of *A. muciniphila* cell-free supernatant on *C. elegans* under a high-glucose diet, especially through the regulation of glucose and lipid metabolisms. The healthy life span of *C. elegans*, the level of free radicals, the activity of antioxidant enzymes, and the related indexes of glucose and lipid metabolisms were detected, and the expression of genes related to glucose and lipid metabolisms was analyzed by real-time fluorescence quantitative PCR. The purpose of this study was to explore the potential of *A. muciniphila* cell-free supernatant as a postbiotic.

## 2. Materials and Methods

### 2.1. Materials and Reagents

*Akkermansia muciniphila* (ATCC BAA-835) was purchased from Beijing Biobw Biotechnology Co., Ltd. (Beijing, China). *C. elegans* (the Bristol strain N2) and *E. coli* OP50 were originally obtained from Caenorhabditis Genetics Center (University of Minnesota, Minneapolis, MN, USA). Sodium chloride, sodium hypochlorite, calcium chloride, and other reagents were purchased from Beijing Luqiao Biological Co., Ltd. (Beijing, China), if not otherwise specified. Glycogen, glucose, triglyceride (TG), and total protein (BCA) assay kits were purchased from Nanjing Jiancheng Bioengineering Institute (Nanjing, China). Catalase (CAT), glutathione peroxidase (GSH-PX), and superoxide dismutase (SOD) kits were purchased from Solarbio Biotechnology Co., Ltd. (Beijing, China) Trizol reagent, HiScript ll Q RT SuperMix kit NXP Biotech Co., Ltd. (Beijing, China) and Power SYBR Green PCR Master Mix were purchased from Vazyme Biotech Co., Ltd. (Nanjing, China)

### 2.2. Sample Preparation

*A. muciniphila* cell-free supernatant: *A. muciniphila* was incubated anaerobically at 37 °C in brain heart infusion supplemented with 0.2% mucin type III until the bacteria reached a late stage of exponential growth. After fermentation, the supernatant was centrifuged at 8000 rpm for 10 min and purified by a 0.22 μm disposable sterile filter membrane. Then the filtrate was distributed and diluted to 2×, 5×, and 10× with sterile water. Finally, the *A. muciniphila* cell-free cultures at different dilutions were stored in a refrigerator at −80 °C until use.

### 2.3. Culture of Caenorhabditis elegans

The *C. elegans* were reared at 20 °C using a standardized method and synchronized three times prior to the experiment [22]. After synchronization, L4 stage nematodes were transferred to fresh NGM plates inoculated with OP50 (containing a final concentration of 0.12 mM 5-fluorodeoxyuridine to inhibit egg hatching). Normal feeding without glucose was used as the control group (C group). The medium was supplemented with glucose (final concentration 2%) for the high-glucose diet feeding group (HG group). Under high-glucose conditions, 100 μL of *A. muciniphila* cell-free supernatant in 2×, 5×, and 10× dilution gradients were applied on the surface of NGM medium, and were defined as the HG + 2×, HG + 5×, and HG + 10× groups, respectively. The *C. elegans* worms were collected after 24 h of intervention to detect the subsequent experimental indices.

### 2.4. Lifespan Analysis

Referring to the previous method with slight modifications [23], *C. elegans* were cultured on 96-well plates, using a total volume of 200 μL of the liquid system, and were divided into five groups (C, HG, HG-2×, HG-5×, and HG-10×). Four replicates of 20 ± 5 *C. elegans* per well were set up in each group, and the transfer date was defined as 0 days. The end of the experiment was recorded as the death of all *C. elegans* in the five groups. Death was deemed to have occurred when the *C. elegans* did not respond to stimulation with strong light and no swallowing movement was recorded. *C. elegans* mortality was observed and scored daily until all worms were dead. Kaplan-Meier was used for the survival condition analysis.

### 2.5. Healthy Lifespan: Head Swings and Pharyngeal Pump Assay

After 24 h of *C. elegans* intervention, the number of head swings and pharyngeal pump swallows in 60 s were observed and recorded by a stereo microscope (Nikon Eclipse E100) equipped with TCapture App video recording software (version 3.9.0.604, Nikon Instruments, Melville, NY, USA). Ten *C. elegans* were counted in each group [24].

### 2.6. Determination of Glycogen and Glucose Content in C. elegans

At the *C. elegans* intervention, the worms were collected with precooled M9 buffer and rinsed three times to wash off the attachment on the surface of the worms. Then the worm was broken by ultrasonic means in an ice water bath to prepare the tissue homogenate. The supernatant was taken for glycogen and glucose assay after centrifugation at 8000 rpm for 10 min. Glycogen and glucose levels in the *C. elegans* were determined according to the kit instructions, and protein concentrations were quantified using the BCA protein assay kit. Three replicates were performed for each group.

### 2.7. Determination of Triglycerides (TG) Content in C. elegans

The level of TG in the tissue homogenate of *C. elegans* was determined by a TG kit, and the quantitative data of the protein concentration was measured by a BCA protein assay kit. Each group was measured three times.

### 2.8. Oil Red O Staining

According to the previous method [25], the *C. elegans* were stained for body fat using Oil Red O. After the intervention, the *C. elegans* were washed with M9 buffer three times, and then the worms were transferred to Oil Red O working solution for staining for 2 h. The dye was then removed by centrifugation, and the worms were washed twice with M9 buffer and resuspended. Finally, 5 μL of worm solution was placed on 2% agarose slides for observation. Ten *C. elegans* were photographed with TCapture software in each group, and the images were analyzed for color density using ImageJ Plus software.

### 2.9. Measurement of Reactive Oxygen Species (ROS)

ROS levels were determined using the fluorescent probe H_2_DCFDA method [26]. The *C. elegans* were collected with M9 buffer and washed three times at the end of the *C. elegans* intervention. The *C. elegans* concentration was then adjusted to 2000 worms/mL using M9 buffer containing 5 μM H_2_DCFDA (Sigma-Aldrich. Louis, MO, USA) and incubated for 3 h protected from light. Then, 50 μL of the worm solution was added to a black 96-well plate. The data were read by a multifunctional enzyme marker (Thermo Scientific™ Multiskan™ FC, Waltham, MA, USA) at the excitation wavelength 485 nm and emission wavelength 530 nm every 10 min for a total time of 2 h. Six assays were performed. The quantified ROS was expressed as an increase in fluorescence in 2 h.

### 2.10. Antioxidant Enzyme Assay

The activities of superoxide dismutase (SOD), glutathione peroxidase (GSH-Px), and catalase (CAT) were determined by a commercial kit method. The supernatant of *C. elegans* tissue homogenate was prepared and normalized for protein concentration for the assay according to the instructions of the kit. Three replicates were set up for each group.

### 2.11. RNA Extraction and qRT-PCR

According to the previous experimental protocol [27], total RNA from *C. elegans* was extracted using the Trizol kit at 4 °C. The purity and concentration of total RNA were determined and analyzed in real time using the Hieff^®^ qPCR SYBR Green Master Mix kit and the Rotor-Gene Q system. Supplementary Material Table S1 lists the key genes and primers involved in glucose metabolism and lipid metabolism. The relative gene expression level was quantified by the CT threshold cycle method. Briefly, we calculated the difference between the average CT value of each sample and the CT value of the internal reference gene (β-action). The results were determined as 2^−ΔΔCT^, where ΔΔC_T_ = ΔC_Tsample_ − ΔC_Tcontrol_ was used as the control. Three replicates were performed for each group.

### 2.12. Statistical Analysis

Differences between groups were calculated using one-way analysis of ANOVA followed by Tukey’s post hoc test for multiple comparisons between more than two groups. All data are expressed as “mean ± standard deviation (SD).” Graphs and analysis were plotted using GraphPad Prism 8.0. In figures, ^#^ *p* < 0.05, ^##^ *p* < 0.01 compared with the control group; * *p* < 0.05, ** *p* < 0.01 compared with the high-glucose group.

## 3. Results

### 3.1. Effect of A. muciniphila Cell-Free Supernatant on Healthy Lifespan of C. elegans

**Figure 1:**
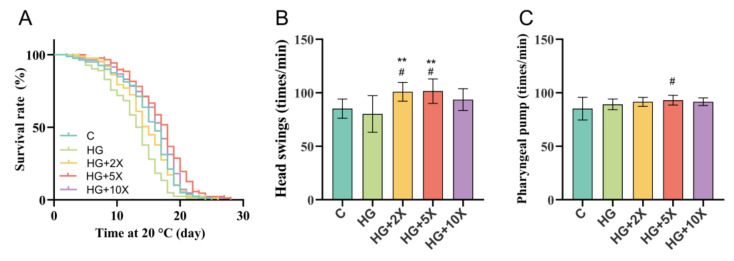
Supplementation with *A. muciniphila* cell-free supernatant on healthy lifespan in *Caenorhabditis elegans.* (**A**) Survival curves; (**B**) head swings; (**C**) pharyngeal pump beating. Results are expressed as mean ± SD deviation, and *p*-values are calculated by Tukey’s test. Compared with the control group, # is significantly different at *p* < 0.05; compared with the high-glucose group, ** is significantly different at *p* < 0.01.

To explore whether *A. muciniphila* cell-free supernatant also has positive effects on other aspects of health, the natural life span and exercise ability of *C. elegans* were evaluated. As shown in Figure 1, *A. muciniphila* cell-free supernatant prolonged the life span and increased the frequency of head swing of *C. elegans*. The average life span of nematodes in the HG group was 12.85 days, which was significantly lower than in the C group. However, the average life span of nematodes in the HG + 5× group was 16.86 d and the longest life span was 28 d, which was higher than that in the HG group (Table 1). Although the HG diet had no significant effect on the frequency and swallowing movement of nematodes, the HG + 2× and HG + 5× groups showed significantly improved head swings ability of the nematodes (*p* < 0.01). Particularly, the HG + 5×-treated significantly improved pharyngeal pump ability of the nematodes (*p* < 0.05). These findings demonstrated that *A. muciniphila* cell-free supernatants contribute to healthy lifespans of *C. elegans.*

### 3.2. Effects of A. muciniphila Cell-Free Supernatant on Glycogen and Glucose Levels of C. elegans

Under a high-glucose diet, the excessive accumulation of glucose and glycogen is the key factor that shortened the life span of *Caenorhabditis elegans* [28]. In order to evaluate the effect of *A. muciniphila* cell-free supernatant on glucose metabolism in nematodes, the contents of glucose and glycogen in each group of *C. elegans* were determined.

**Figure 2:**
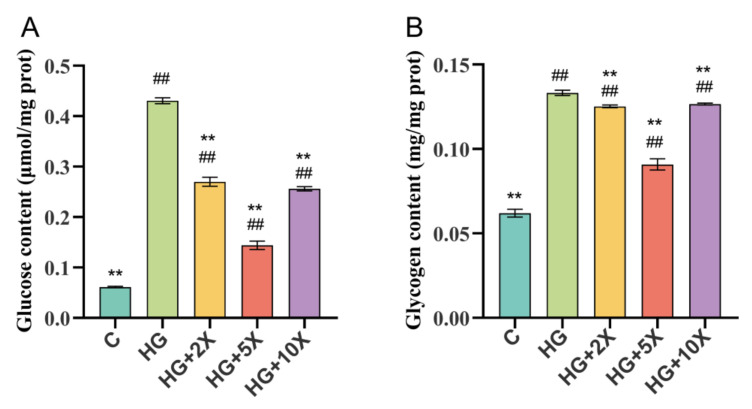
Effect of supplementation with *A. muciniphila* cell-free supernatant on glucose and glycogen content in *C. elegans*. (**A**) Glucose content; (**B**) glycogen content. Results are expressed as mean ± SD deviation, and *p*-values are calculated by Tukey’s test. Compared with the control group, ## is significantly different at *p* < 0.01; compared with the high-glucose group, ** is significantly different at *p* < 0.01.

As expected, the high-glucose treatment significantly increased the glucose and glycogen levels in *C. elegans* compared with the normal treatment (*p* < 0.01) (Figure 2). *A. muciniphila* cell-free supernatant supplementation effectively alleviated the high-glucose-induced increase in the contents of glucose and glycogen in nematodes, especially in the HG + 5× group compared to the HG group, which decreased by 66.6% and 31.8% in glucose and glycogen content, respectively. Thus, our results suggested that *A. muciniphila* cell-free supernatant could reduce glycogen and glucose in *C. elegans*.

### 3.3. Effect of A. muciniphila Cell-Free Supernatant on the Level of TG in C. elegans

**Figure 3:**
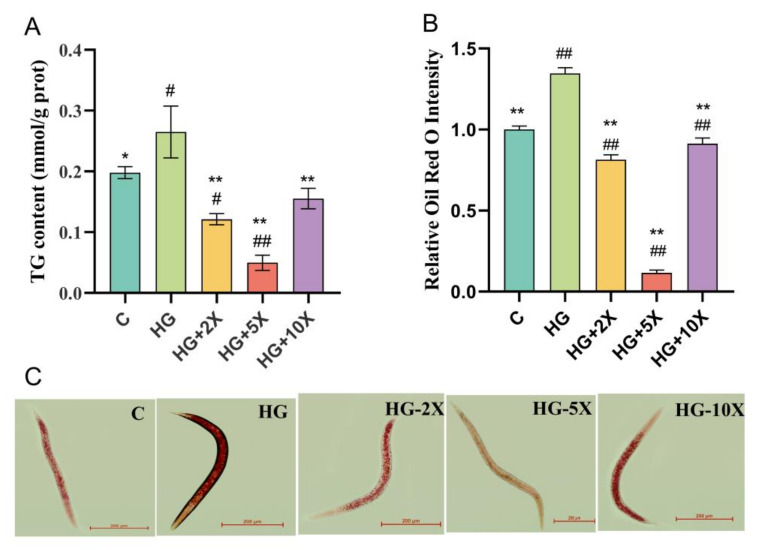
Effect of supplementation with *A. muciniphila* cell-free supernatant on lipid metabolism in *C. elegans.* (**A**) TG content; (**B**) Quantification of data shown in (**C**). N = 10. (**C**) Oil Red O staining nematode fat and selected intuitive characteristics map. Bar, 200 µm. Results are expressed as mean ± SD deviation, and *p*-values are calculated by Tukey’s test. Compared with the control group, # and ## are significantly different at *p* < 0.05 and *p* < 0.01, respectively; compared with the high-glucose group, * and ** are significantly different at *p* < 0.05 and *p* < 0.01, respectively.

Generally, lipids are stored as lipid droplets in the cells of the intestine and subcutaneous tissue in *C. elegans* [29]. In order to explore the effect of *A. muciniphila* cell-free supernatant on lipid metabolism in *C. elegans*, the triglyceride content was evaluated. Staining live worms with the lipophilic dye Oil Red O revealed differences in staining intensity in the worms. High glucose induced the significant increase of triglyceride content in the nematodes, but *A. muciniphila* cell-free supernatant could inhibit this change (Figure 3A). The content of triglyceride in nematodes in the HG + 5× group was the lowest, which decreased by 81.2% compared with the HG group. The results of Oil Red O staining were consistent with those of TG (Figure 3B,C). High glucose induced the wide and dense distribution of nematode lipid droplets, but the lipid droplet density decreased under the intervention of *A. muciniphila* cell-free supernatant. Therefore, *A. muciniphila* cell-free supernatant can reduce the level of triglyceride and fat accumulation in *C. elegans*.

### 3.4. Effects of A. muciniphila Cell-Free Supernatant on Levels of Free Radicals and Antioxidant Enzymes in C. elegans

**Figure 4:**
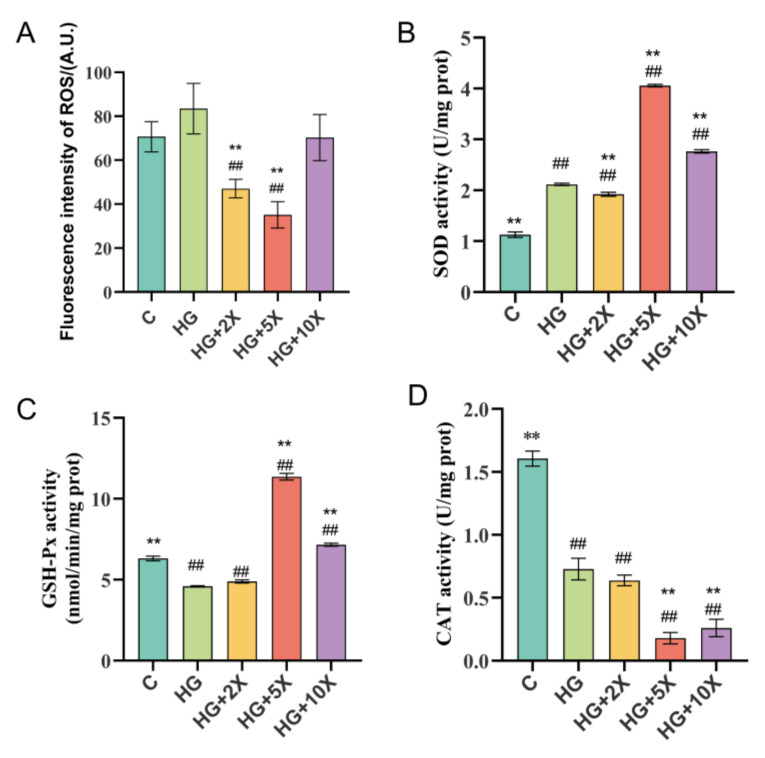
Effect of supplementation with *A. muciniphila* cell-free supernatant on antioxidant stress in *C. elegans*. (**A**) ROS fluorescent labeling test results; (**B**) SOD activity; (**C**) GSH-Px activity; (**D**) CAT activity. Results are expressed as mean ± SD deviation, and *p*-values are calculated by Tukey’s test. Compared with the control group, ## is significantly different at *p* < 0.01; compared with the high-glucose group, ** is significantly different at *p* < 0.01.

Excessive production of ROS can lead to excessive accumulation of fat, and hydroxyl free radicals have destructive effects on the structures of proteins and lipids [30]. We next investigated whether *A. muciniphila* cell-free supernatant supplementation could improve antioxidant stress in *C. elegans*. The ROS level was significantly increased by high glucose consumption, which was attenuated by *A. muciniphila* cell-free supernatant supplementation (Figure 4A). Furthermore, the *A. muciniphila* cell-free supernatant supplementation led to an increase in SOD and GSH-Px levels, as well as a decrease in the CAT level (Figure 4B–D). The activities of SOD and GSH-Px in the HG + 5× group compared with the HG group were increased by 47.83% and 59.64%, respectively. Therefore, *A. muciniphila* cell-free supernatant may suppress oxidant stress by decreasing the CAT level and promoting SOD and GSH-Px activities in *C. elegans*.

### 3.5. Effects of A. muciniphila Cell-Free Supernatant on Glucose and Lipid Key Gene Expression in C. elegans

The above results indicate that *A. muciniphila* cell-free supernatant had a positive effect on the health of *C. elegans*. It was able to promote the metabolism of glucolipids and reduce the storage of carbohydrate substances and fat in *C. elegans*. Next, we explored the mechanism of the effect of *A. muciniphila* cell-free supernatant on glucolipid metabolism in *C. elegans*. QRT-PCR was used to detect the expression of genes encoding key enzymes in glucolipid metabolism.

**Figure 5:**
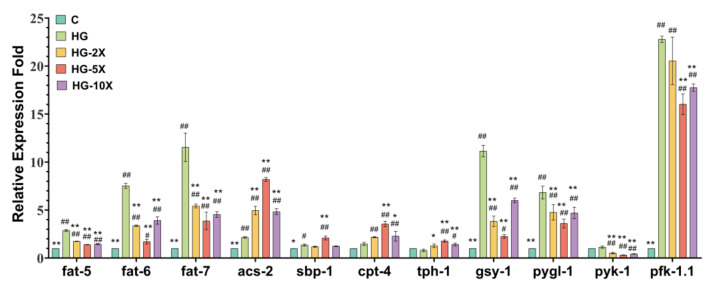
Supplementation with *A. muciniphila* cell-free supernatant on glycolipid metabolism gene expression of *C. elegans*. The results are expressed as mean ± SD deviation, and *p*-values are calculated by Tukey’s test. Compared with the control group, # and ## are significantly different at *p* < 0.05 and *p* < 0.01, respectively; compared with the high-glucose group, * and ** are significantly different at *p* < 0.05 and *p* < 0.01, respectively.

According to Figure 5, the *gsy-1*, *pygl-1*, *pfk-1.1*, *pyk-1*, *fat-5*, *fat-6*, and *fat-7* genes were downregulated, while the *acs-2*, *cpt-4*, *sbp-1,* and *tph-1* genes were upregulated with *A. muciniphila* cell-free supernatant supplementation. The gene *acs-2* is a key gene in the initiation of fatty acid β oxidation, and *pyk-1* is a gene encoding pyruvate kinase, which is the most important gene in the biological process of glycolipid metabolism. Compared with the HG group, the expression of the *acs-2* gene was upregulated 3.80-fold, and *pyk-1* was downregulated by 72.30% in the HG + 5× group. Hence, *A. muciniphila* cell-free culture supernatant may inhibit glycolysis and promote fatty acid β oxidation to reduce fat accumulation in *C. elegans*.

## 4. Discussion

*A. muciniphila* plays a significant role in regulating glucose and lipid metabolism. *A. muciniphila* secretes short-chain fatty acids (SCFAs) to promote intestinal microbial homeostasis and protect intestinal barrier function [31]. The SCFAs secreted by *A. muciniphila* include acetic acid, propionic acid, and isovaleric acid. These SCFAs have been shown to provide energy for colon cells, improve ion absorption, have anti-inflammatory properties, and regulate the production of serotonin [32]. It has also been reported that SCFAs such as butyrate play a role in regulating neurological function [33]. However, when SCFAs act on adipose tissue and other peripheral tissues, they must go through systemic circulation, and some of them are used to play a targeted role. The estimated concentration is in the range of micromoles [34]. Therefore, in this experiment, different dilutions of *A. muciniphila* cell-free supernatant were used to interfere with the nematodes in order to explore the biological effects on the nematodes, and preliminarily discuss the action targets.

The intestinal microflora contribute to the absorption of carbohydrates and lipids, and *A. muciniphila* plays an important role in energy metabolism [35]. The results of this study showed that the contents of glucose, glycogen, and triglyceride in nematodes fed with *A. muciniphila* acellular supernatant decreased significantly. It can be inferred that the metabolites of *A. muciniphila* have similar functions to *A. muciniphila*. According to the principle that an increase in heat production and energy consumption is usually accompanied by an increase in metabolic rate [36], we hypothesized that the decrease in glucose, glycogen, and triglycerides in the nematodes may be due to metabolites in the supernatant of *A. muciniphila* culture that promote energy conversion. This is consistent with the conclusion that the movement of nematodes was accelerated. Meanwhile, *A. muciniphila* cell-free supernatant prolonged the healthy life of *C. elegans*, which is very similar to the results of previous studies on mammals with *A. muciniphila* bacteria [37].

Previous studies have shown that excessive accumulation of nutrients such as carbohydrates and lipids can lead to an increase in the production of ROS, which leads to oxidative stress and even to mitochondrial dysfunction [38,39]. However, excessive production of ROS can also induce excessive fat accumulation [30]. In the current study, the acellular supernatant of *A. muciniphila* decreased the ROS level of *C. elegans* and increased the activities of SOD and GSH-Px. As antioxidant enzymes can protect organisms from oxidative stress, the decrease of ROS content in this study may be related to the increase of antioxidant enzymes. This suggests that the antioxidative effect of the acellular supernatant of *A. muciniphila* can help nematodes reduce fat accumulation.

We proved that *A. muciniphila* cell-free supernatant can improve the glycolipid metabolism of nematodes, but the key question is how *A. muciniphila* cell-free supernatant plays this role. To further clarify the action pathway of *A. muciniphila* supernatant, the changes of key genes in the process of glucose and lipid metabolisms were detected. Endogenous glucose in organisms is mainly produced by glycogen decomposition and gluconeogenesis. The reaction is mainly catalyzed by glycogen synthase (encoded by *gsy-1* gene) and glycogen phosphorylase (encoded by *pygl-1* gene). The results showed that the cell-free supernatant of *A. muciniphila* reduced the expression of *gsy-1* and *pygl-1*, which indicated that the cell-free supernatant of *A. muciniphila* can reduce the total glucose by inhibiting the decomposition of glycogen. Fructose-6-phosphate kinase (encoded by *pfk-1.1* gene) and pyruvate kinase (encoded by *pyk-1* gene) are rate-limiting enzymes in glycolysis and key enzymes in the process of glycolipid conversion. The results showed that the acellular supernatant of *A. muciniphila* downregulated the expression of *pfk-1.1* and *pyk-1*. However, the level of glucose in the nematode did not increase. It is speculated that the reason for this may be that *A. muciniphila* cell-free supernatant can inhibit the diet of *C. elegans*, so that the food intake of the nematodes is reduced in addition to the glucose that permeates into the body. The energy generated by glucose oxidation may not be sufficient to support the life activities of nematodes, thus they consume a large amount of glucose to convert into fatty acids, thereby increasing the level of β-oxidation and generating energy to support life activities.

In lipolysis, the degradation of fatty acids requires the activation of fatty acid CoA synthetase encoded by the *acs-2* gene, to catalyze the synthesis of fatty acyl CoA. Then, carnitine palmitoyltransferase I, encoded by the *cpt-4* gene, enters the mitochondria for β-oxidative decomposition [40]. In the above process, *acs-2* and *cpt-4* are the key genes that induce fatty acids to enter β-oxidation. Hence, the upregulation of these two genes can be used as a sign of enhanced fatty acid β oxidation. In addition, the overexpression of the *acs-2* gene can push *Caenorhabditis elegans* into a low-fat state [41], which further indicates that it is a key target gene in *A. muciniphila* cell-free supernatant to regulate lipid metabolism. Previous studies have shown that some natural products can reduce the total fat content of *C. elegans* by upregulating the *acs-2* gene [42]. This provides a theoretical basis for the above hypothesis. The high homology of cholesterol regulatory element binding protein (SREBPs) in mammals is closely related to the syntheses and metabolisms of cholesterol, triglycerides, and fatty acids [43]. The SBP-1 of *C. elegans* is highly homologous to SREBPs, so the *sbp-1* gene plays a key role in lipid metabolism. In another study, RNA interference (RNAi) of *fat-7* was found to reduce fat content and increase the expression of genes related to β oxidation, including *acs-2* [44]. This shows that *fat-7* can regulate β-oxidation through a negative feedback mechanism. Our results showed that *A. muciniphila* acellular supernatant can upregulate the expression of the *sbp-1* gene in *Caenorhabditis elegans*. Additionally, it downregulates the *fat-7* gene, reduces fat synthesis, and reduces the content of total fatty acids in nematodes. Tph-1 is an important gene that induces the serotonin pathway in *C. elegans*. In the absence of *tph-1* and serotonin, it can lead to an increase in fat deposition, a decrease in fecundity, and a decrease in the metabolic rate in nematodes [45]. The results of this study showed that *A. muciniphila* cell-free supernatant upregulated the expression of the *tph-1* gene. This may also be one of the reasons for the decrease in the total fat content of the nematodes.

**Figure 6:**
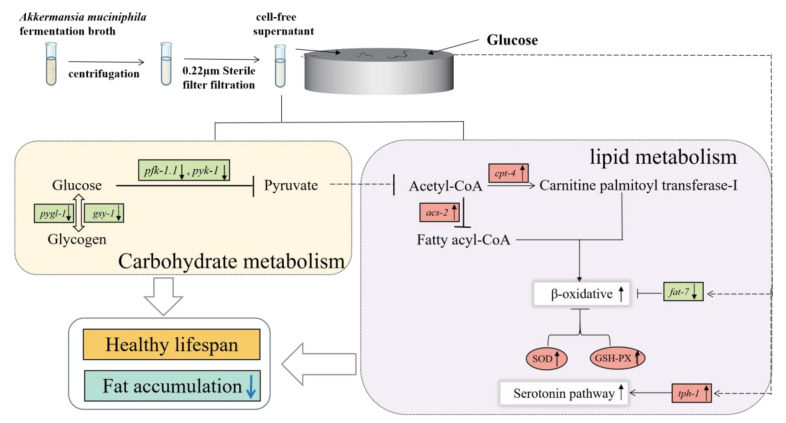
Molecular mechanisms by which *A. muciniphila* cell-free supernatant improves glucolipid metabolism in *C. elegans*. “↑” in boxes represent upregulation, and “↓” in boxes represent downregulation. Other are connecting lines.

Together, these findings suggest that *A. muciniphila* cell-free supernatant can reduce the levels of glucose and triglycerides in *C. elegans*. The possible mechanism is inhibition of glucose metabolism by downregulating the expression of the *pygl-1*, *gsy-1*, *pfk-1.1,* and *pyk-1* genes, and increasing the level of β-oxidation and fatty acid decomposition by upregulating the expressions of the *acs-2* and *cpt-4* genes. At the same time, the expression of the *fat-7* gene is downregulated to reduce fat synthesis (Figure 6).

## 5. Conclusions and Perspective

In conclusion, our results indicated that *A. muciniphila* cell-free supernatant can reduce ROS levels by inhibiting oxidative stress in nematodes, as well as improve glucose and lipid metabolisms, and thus support the healthy life of *C. elegans*. However, there are some limitations to this study. Although different concentrations of *A. muciniphila* cell-free supernatant can improve glucose and lipid metabolisms, significant changes in glucose and lipid metabolisms characteristics, such as glucose and triglyceride levels, also occurred. Different dilution ratios led to dose differences in the active components of *A. muciniphila* cell-free supernatant, but the specific components were determined by GC-MS detection and analysis. Another potential limitation is that although *A. muciniphila* cell-free supernatant has been preliminarily investigated for regulating glycolysis pathways, beta oxidation pathways, and serotonin pathways to control fat accumulation, it has not been properly validated for key targets. In the future, RNA interference technology should be used to knock out or silence key genes in several pathways to detect the specific roles of these genes in lipid deposition and conduct a more comprehensive study and analysis of the effective mechanism of *A. muciniphila* cell-free supernatant to improve lipid metabolism. Therefore, this study provides new insights into the potential of adding *A. muciniphila* postbiotics to food substrates or health products for the prevention of obesity.

## Figures and Tables

**Table 1:** Effects of supplementation with *A. muciniphila* cell-free supernatant on life span in *Caenorhabditis elegans*.

Group	Number of Worms	Mean Lifespan (Days)	Maximum Lifespan (Days)	Median (Days)
C	79	15.10 ± 0.50	25.00	16.00
HG	82	12.85 ± 0.47	24.00	13.50
HG + 2×	87	14.61 ± 0.46	26.00	15.00
HG + 5×	87	16.86 ± 0.48	28.00	18.00
HG + 10×	83	15.92 ± 0.50	28.00	17.00

## Data Availability

Data is contained within the article or Supplementary Material.

## Supplementary Materials

The following supporting information can be downloaded at: https://www.mdpi.com/article/10.3390/nu15071725/s1, Table S1: Primer sequences for qRT-PCR.
