# Supplementary material for: Akkermansia muciniphila Cell-Free Supernatant Improves Glucose and Lipid Metabolisms in Caenorhabditis elegans
Source: Nutrients. 2023 Mar 31;15(7):1725. doi: 10.3390/nu15071725 (PMC10097305; doi:10.3390/nu15071725)
Supplement: Supplementary file 1 [file nutrients-15-01725-s001.zip › nutrients-2319401-supplementary.pdf]

Table S1. Primer sequences for qRT-PCR.

Genes	Forward sequences (5′-3′)	Reverse sequences (5′-3′)
β-actin	GCCGGAGACGACGCTCCACGCG	GCCTCGTCTCCGACGTACGAGTC
gsy-1	ATCCTCGGATTCATGGTCGC	GCTCCACCAGCACACAAATG
pygl-1	CCTCATCGTCGAGTCCGAAG	GTAGTAGTCGCGATCGGTGG
pfk-1.1	TATGGTCCGTGTGCCACTTC	TCTCTGGAAACTGCGTCCAC
pyk-1	TCAGGTACAAGACGTCGAGC	CCAGCATGCTCCTCTTCGAT
acs-2	GCAGCCTCGCTCTACACTCT	GACTCCTGCAAATGCACATGC
sbp-1	CACCACCTCATCACCACCATCAC	TTCGTCTCTGGAGCATCTTCAATCG
cpt-4	TGACAATCGCACCTCCACAG	TGAATGCTTCGTCCCTCGTT
tph-1	CGGTGAGCCAATTCCGCGAA	AGAACTGCTTGCATGCGTGC
ech-1.1	CGTCGGAGCTGGATTCATGG	TCGTTCAACGCCTGCCTGGT
fat-5	CGGCCGCCCTCTTCCGTTAC	TGGCTGCCATCCGACCCAGT
fat-6	TCAACAGCGCTGCTCACTAT	TTCGACTGGGGTAATTGAGG
fat-7	CAACAGCGCTGCTCACTATT	CACCAACGGCTACAACTGTG
